# Comparative Analysis of Bacterial Information of Biofilms and Activated Sludge in Full-Scale MBBR-IFAS Systems

**DOI:** 10.3390/microorganisms12061121

**Published:** 2024-05-31

**Authors:** Xiaolin Zhou, Haicheng Liu, Xing Fan, Xuyi Wang, Xuejun Bi, Lihua Cheng, Shujuan Huang, Fangchao Zhao, Tang Yang

**Affiliations:** State and Local Joint Engineering Research Centre of Urban Wastewater Treatment and Reclamation, Qingdao University of Technology, Qingdao 266520, Chinazfc870416@163.com (F.Z.); tyang37@163.com (T.Y.)

**Keywords:** biofilm, activated sludge, nitrogen metabolism, bacterial community, potential function

## Abstract

This study extensively analyzed the bacterial information of biofilms and activated sludge in oxic reactors of full-scale moving bed biofilm reactor-integrated fixed-film activated sludge (MBBR-IFAS) systems. The bacterial communities of biofilms and activated sludge differed statistically (R = 0.624, *p* < 0.01). The denitrifying genera *Ignavibacterium*, *Phaeodactylibacter*, *Terrimonas*, and *Arcobacter* were more abundant in activated sludge (*p* < 0.05), while comammox *Nitrospira* was more abundant in biofilms (*p* < 0.05), with an average relative abundance of 8.13%. *Nitrospira* and *Nitrosomonas* had weak co-occurrence relationships with other genera in the MBBR-IFAS systems. Potential function analysis revealed no differences in pathways at levels 1 and 2 based on the Kyoto Encyclopedia of Genes and Genomes (KEGG) between biofilms and activated sludge. However, in terms of pathways at level 3, biofilms had more potential in 26 pathways, including various organic biodegradation and membrane and signal transportation pathways. In comparison, activated sludge had more potential in only five pathways, including glycan biosynthesis and metabolism. With respect to nitrogen metabolism, biofilms had greater potential for nitrification (ammonia oxidation) (M00528), and complete nitrification (comammox) (M00804) concretely accounted for methane/ammonia monooxygenase (K10944, K10945, and K10946) and hydroxylamine dehydrogenase (K10535). This study provides a theoretical basis for MBBR-IFAS systems from the perspective of microorganisms.

## 1. Introduction

The discharge standard of treated wastewater into surface water is tightening, especially for nitrogen [1,2]. However, some conventional technologies for nutrient removal have faced challenges in achieving the required efficiency [1]. Among these technologies, the activated sludge process for biological nitrogen removal was generally applied in existing wastewater treatment plants (WWTPs) because of its economic advantages [3]. Therefore, an effective method for upgrading activated sludge to improve nitrogen removal efficiency is imperative to meet the increasingly stringent discharge standard [3]. Thereby, moving bed biofilm reactor-integrated fixed-film activated sludge (MBBR-IFAS) technology was introduced to WWTPs to upgrade them worldwide [4,5,6]. In recent years, this process has become a leading option for treating wastewater [7]. It combines the benefits of biofilms and activated sludge to improve pollutant removal capacities and reduce the risk of washing away slow-growing microorganisms [4,7].

Microbial communities are thought to affect the efficiencies and stabilities of wastewater treatment systems [8]. In recent years, studies have been carried out to research the microbial communities of MBBR-IFAS systems. For example, the bacterial community information of temporal samples in MBBR-IFAS systems was investigated in detail by varying sludge retention times [9]; the effects of dissolved oxygen (DO) on microbial communities in MBBR-IFAS systems for simultaneous nitrification and denitrification were researched [4]; and the microbial communities for partial nitrogen/anammox in MBBR-IFAS systems were studied [10]. However, most of these studies were based on lab-scale or pilot-scale experiments, focusing on the microbial communities of MBBR-IFAS systems that are affected by various factors or have special functions. Little attention has been paid to the microbial information of full-scale running municipal MBBR-IFAS systems. In-depth exploration of the microbial information of full-scale, running MBBR-IFAS systems is crucial to understanding MBBR-IFAS systems better for practical operation. Moreover, biofilms and activated sludge coexist in MBBR-IFAS systems in attachment and flocs, respectively, and they are two common microbial communities with distinctive properties [7]. Comparative analysis of bacterial information between biofilms and activated sludge simultaneously in MBBR-IFAS systems is of great importance in providing insight into their roles in pollutant removal.

For some time now, Phylogenetic Investigation of Communities by Reconstruction of Unobserved States (PICRUSt2) matched up with high-throughput sequencing has been extensively used in predicting microbial functions based on 16S rRNA gene sequencing [11]. This method has increased accuracy due to the excellent coverage of the taxonomic diversity of the reference genome database in recent years [12,13,14]. PICRUSt2 is an effective method for gaining an in-depth understanding of the functional roles of microbial communities in various environments [15].

Therefore, in this study, one typical municipal WWTP (250,000 m^3^/d) was selected to explore and comparatively analyze the bacterial community diversity and bacterial populations of biofilms and activated sludge by high-throughput sequencing. Moreover, the bacterial co-occurrence network was studied. Specially, this plant contains three independent MBBR-IFAS systems with the same influent water and climate conditions, providing the conditions for parallel investigations. In addition, the potential functions based on the Kyoto Encyclopedia of Genes and Genomes (KEGG) of the biofilms and activated sludge in MBBR-IFAS systems were explored by PICRUSt2. This research aims to understand MBBR-IFAS systems in depth from the perspective of microorganisms and provide the theoretical basis for its practical application to obtain high efficiency in biological wastewater treatment.

## 2. Materials and Methods

### 2.1. Treatment Process, Sewage Characteristic, and Sample Collection

**Figure 1:**
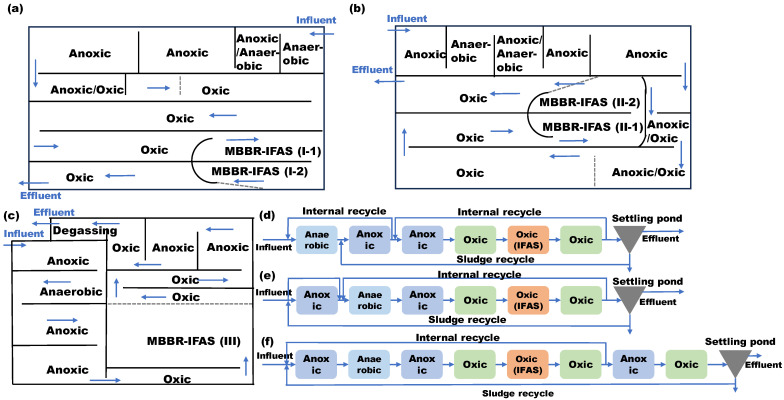
The layout diagrams of Systems I (**a**), II (**b**), and III (**c**) and flow diagrams of Systems I (**d**), II (**e**), and III (**f**).

The samples in this study were collected in a municipal WWTP in the Shandong Province of China. The total treatment capacity and personal equivalent (PE) of this plant were 250,000 m^3^/d and 3,000,000, respectively. This WWTP includes three independent secondary biological treatment systems, numbered Systems I, II, and III. The capacities of Systems I, II, and III were 95,000 m^3^/d, 110,000 m^3^/d, and 45,000 m^3^/d, respectively. The layout and flow diagrams of Systems I, II, and III are shown in Figure 1. Systems I, II, and III adopted an improved University of Cape Town (UCT) process [16], an improved anaerobic/anoxic/oxic (A/A/O) process [17], and the Bardenpho process [18], respectively. The month’s influent and effluent water qualities of this plant during sample collections were as follows: five-day biochemical oxygen demand (BOD_5_) concentrations were 369 mg/L and 4.24 mg/L; chemical oxygen demand (COD) concentrations were 818 mg/L and 20 mg/L; suspended solids (SS) concentrations were 568 mg/L and 5 mg/L; ammonia nitrogen (NH_3_-N) concentrations were 48.39 mg/L and 1.35 mg/L; total nitrogen (TN) concentrations were 77.31 mg/L and 11.01 mg/L; and total phosphorus (TP) concentrations were 10.95 mg/L and 0.10 mg/L. The effluent water satisfied the First-order Class-A of Chinese Discharge Standard of Pollutants for Municipal WWTP Discharge Standard (GB18918-2002). The high-density polyethylene suspended carriers (SPR-I, 450 m^2^/m^3^ and SPR-II, 620 m^2^/m^3^) were added to some aeras of oxic reactors with a filling fraction of ~40% so that the systems could achieve the required ammonia nitrogen removal capacities. All the carriers were retained in the systems by the perforated sieves on the effluent end of the reactors, but the activated sludge could pass through to the next unit with the treated water [19].

For System I, with a total of four oxic galleries, the suspended carriers were added to the tail of the third oxic zone (the first MBBR-IFAS of System I) and the head of the fourth oxic zone (the second MBBR-IFAS of System I); for System II, with a total of three oxic zones, the suspended carriers were added to the tail of the second oxic zone (the first MBBR-IFAS of System II) and the head of the third oxic zone (the second MBBR-IFAS of System II); for System III, the suspended carriers were added to the first-stage oxic reactors (MBBR-IFAS of System III). So, these areas formed MBBR-IFAS systems. The biofilms attached to the suspended carriers and activated sludge were collected randomly from these MBBR-IFAS systems. These samples were numbered as shown in Table 1.

### 2.2. High-Throughput Sequencing Methods

The biofilms obtained for high-throughput sequencing were scratched from the suspended carriers. The activated sludge mixture was centrifuged at 3000 r/min, and the supernatant was poured off. Then, the biofilms and activated sludge were stored at −80 °C immediately. Deoxyribonucleic acid (DNA) extraction and sequencing were entrusted to Yuanxu Biotechnology Co., Ltd. (Shanghai, China). The detailed procedures were performed on the Illumina Miseq platform (paired-end, 2 × 300) following the manufacturer’s protocols [20,21]. The V3-V4 region of the bacterial 16S rRNA gene was amplified using universal primers 338F and 806R.

### 2.3. Potential Function Analysis

The total sequencing reads were annotated from the KEGG databases (http://www.genome.jp/kegg/, accessed on 1 December 2023 and 1 January 2024) by means of PICRUSt2 to predict gene functions [11,13,14]. This part of the analysis was performed using the Majorbio Cloud Platform online tool (https://cloud.majorbio.com/page/tools/, accessed on 1 December 2023) [22].

### 2.4. Ammonia Nitrogen Removal Capability Test

After the carriers and activated sludge were sampled from the three MBBR-IFAS systems, they were added to six reactors with a volume of 5 L, respectively. Then, the secondary sedimentation tank effluent water was added to the scale mark. The filling fraction of the suspended carriers was 40%, and the mixed liquid suspended sludge of activated sludge was ~6000 mg/L, corresponding to the full-scale systems. The initial ammonia nitrogen concentration of ~30 mg N/L was obtained by adding ammonium chloride into the reactor. At this moment, air was added to these reactors simultaneously, and the clock started. During the operation, the DO concentration was controlled at 2–4 mg/L. The test lasted three hours, and water samples were taken every 30 min. The ammonia nitrogen concentration was finally tested according to the standard method [23,24].

### 2.5. Statistical Analysis

The difference analysis and co-occurrence network analysis in this study were performed using an online gene cloud tool (https://www.genescloud.cn, accessed on 1 December 2023 and 1 January 2024), with the significance level *p*-value set at 0.05.

## 3. Results and Discussion

### 3.1. Bacterial Community Diversity

#### 3.1.1. Alpha Bacterial Community Diversity

**Figure 2:**
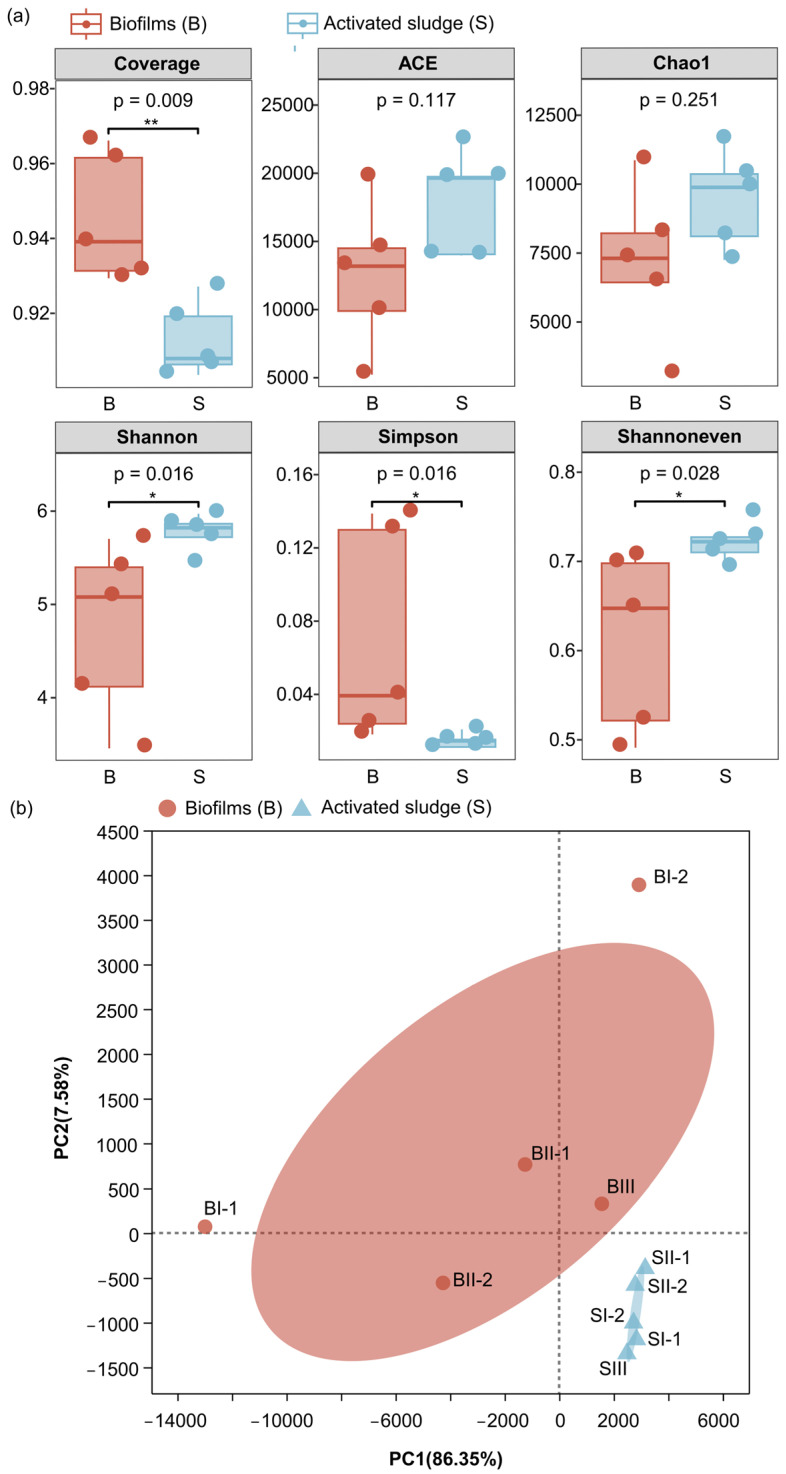
Difference analysis of alpha diversity indexes between biofilms and activated sludge in MBBR-IFAS systems (**a**) and the PCA for the bacterial communities in MBBR-IFAS systems (**b**). “*” presents “*p* < 0.05”, and “**” presents “*p* < 0.01”.

The alpha diversities of biofilms and activated sludge in MBBR-IFAS systems are shown in Figure 2a. The coverage of all biofilms and activated sludge samples was above 90%, indicating that sequencing data give an accurate picture of the bacterial community. However, there may still be a small number of microbial populations that remain undetected. Abundance-based coverage estimator (ACE) and Chao1 indexes represented community richness [25,26]. ACE indexes of biofilms and activated sludge were 12,492 ± 5380 and 17,955 ± 3786, respectively, and their Chao1 indexes were 7185 ± 2822 and 9438 ± 1755, respectively. No significant differences were found in bacterial community richness between biofilms and activated sludge in MBBR-IFAS systems (*p* > 0.05). A larger Shannon index value and a smaller Simpson index value indicate more community diversity [27,28,29]. The Shannon indexes of biofilms and activated sludge were 4.75 ± 0.94 and 5.76 ± 0.02, respectively, and their Simpson indexes were 0.070 ± 0.059 and 0.014 ± 0.004, respectively. Therefore, significantly higher bacterial community diversity was found in activated sludge than in biofilms (*p* < 0.05). This may be related to biofilms always being in a relatively stable environment compared to activated sludge. Concretely, in these systems in the study, the activated sludge circulated in anaerobic, anoxic, and oxic reactors, while biofilms always remained in oxic reactors. Therefore, microorganisms in activated sludge adapted to various conditions, and microorganisms in biofilms adapted to specific aerobic conditions. The Shannoneven index indicates community evenness [30]. The values of biofilms and activated sludge were 0.61 ± 0.10 and 0.72 ± 0.02, meaning that significantly higher community evenness was found in activated sludge than in biofilms (*p* < 0.05).

In conclusion, biofilms had higher bacterial community diversity than activated sludge in MBBR-IFAS systems, and no significant differences were found in community richness and evenness between them.

#### 3.1.2. Beta Bacterial Community Diversity

The principal component analysis (PCA) revealed beta diversity in bacterial communities (Figure 2b). P1 and P2 explained 86.35% and 7.58% variance in bacterial communities, respectively. As a result, the samples are divided into two groups: biofilm and activated sludge samples. The analysis of similarities (ANOSIM) confirms that the grouping is statistically significant (R = 0.624, *p* < 0.01).

In conclusion, there were different bacterial communities between biofilms and activated sludge in MBBR-IFAS systems.

### 3.2. Bacterial Populations

#### 3.2.1. Bacterial Populations at Phylum Level

**Figure 3:**
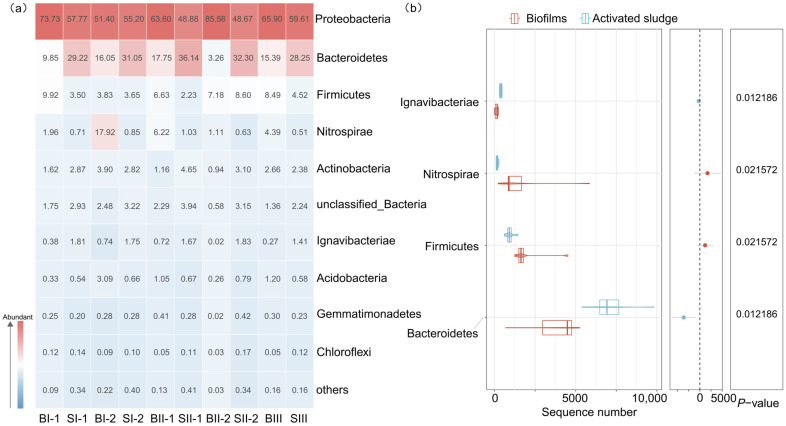
Relative abundance of the biofilms and activated sludge (**a**) and difference analysis between biofilms and activated sludge (**b**) in MBBR-IFAS systems at the phylum level.

As depicted in Figure 3a, ten kinds of bacterial populations at the phylum level exceed 0.1%. Proteobacteria was the most abundant phylum in biofilms and activated sludge systems in MBBR-IFAS systems. This phylum has always been reported as the main dominant phylum in WWTPs [31]. The relative abundance of the samples was 48.67–85.56%. The next most abundant phylum in both biofilms and activated sludge was Bacteroidetes, and it was also the main dominant phylum in WWTPs [32,33]. However, the relative abundance of Bacteroidetes in biofilms and activated sludge was 12.46% ± 5.94% and 31.39% ± 3.09%, respectively, and this phylum was statistically more abundant in activated sludge than that in biofilms (*p* < 0.05) (Figure 3b). It was reported that Bacteroidetes were related to the hydrolysis of macromolecules under anaerobic or anoxic conditions [31,34]. Among the top ten abundant phyla in MBBR-IFAS systems, Ignavibacteriae was also statistically more abundant in activated sludge than in biofilms (*p* < 0.05). Ignavibacteriae was reported to be related to cellulolytic [35]. Firmicutes and Nitrospirae were statistically more abundant in biofilms than in activated sludge (*p* < 0.05). Firmicutes were reported to be the dominant bacteria in antibiotic pharmaceutical wastewater [36,37,38]. Nitrospirae is highly correlated with nitrification efficiency [32,39].

#### 3.2.2. Bacterial Populations at Genus Level

**Figure 4:**
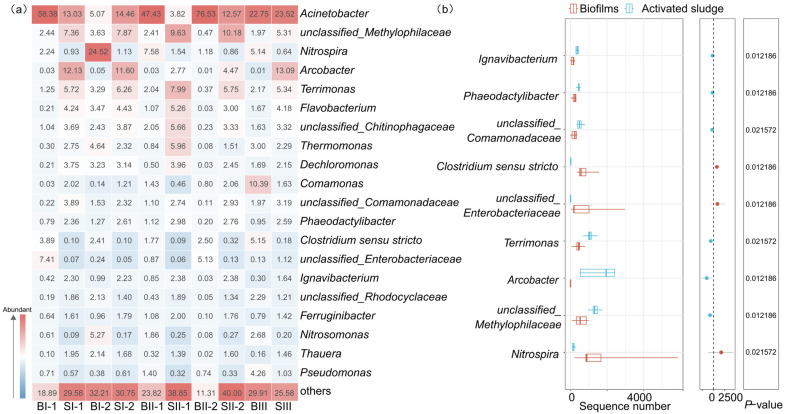
Relative abundance of the biofilms and activated sludge (**a**) and difference analysis between biofilms and activated sludge (**b**) in MBBR-IFAS systems at the genus level.

As depicted in Figure 4a, twenty kinds of bacterial populations at the genus level exceed 1%. The most abundant genus in MBBR-IFAS systems was *Acinetobacter*. The average relative abundance of *Acinetobacter* was 27.76%. This genus was reported to be aerobic denitrifier [40,41], suggesting denitrification occurred in the oxic reactors. Moreover, *Arcobacter*, *Terrimonas*, *Flavobacterium*, *Thermomonas*, *Dechloromonas*, *Comamonas*, *Phaeodactylibacter*, *Clostridium sensu stricto*, *Ignavibacterium*, *Ferruginibacter*, *Thauera*, and *Pseudomonas* have all been reported to be related to denitrification [42,43,44,45,46,47,48]. Among them, *Ignavibacterium*, *Phaeodactylibacter*, *Terrimonas*, and *Arcobacter* were more significantly abundant in activated sludge than in biofilms (*p* < 0.05) (Figure 4b). Correspondingly, activated sludge flew into the anoxic reactors for denitrification, while the biofilms were always retained in the oxic reactors. *Nitrospira*, regarded as comammox bacteria [49], was the third abundant genus in MBBR-IFAS systems. The average relative abundances of *Nitrospira* in biofilms and activated sludge were 8.13% and 1.02%, respectively. *Nitrospira* was significantly more abundant in biofilms than activated sludge (*p* < 0.05). It was investigated that the average abundance of *Nitrospira* in activated sludge of 16 WWTPs was 2.8% [33], higher than the activated sludge but much lower than the biofilms of MBBR-IFAS systems. Overall, *Nitrospira* was enriched in the MBBR-IFAS systems because of biofilms. In addition, *Clostridium sensu stricto* was also significantly more abundant in biofilms than activated sludge (*p* < 0.05). This genus was reported to be dominant in a continuous aeration system [46], suggesting that its enrichment in biofilms was caused by the biofilms always being in aerobic conditions. *Nitrosomonas*, another genus associated with nitrification, was found in MBBR-IFAS systems with an average abundance of 1.15%.

In summary, the unique environments of biofilms and activated sludge in MBBR-IFAS systems inevitably form their unique bacterial communities.

### 3.3. Bacterial Co-Occurrence Network

**Figure 5:**
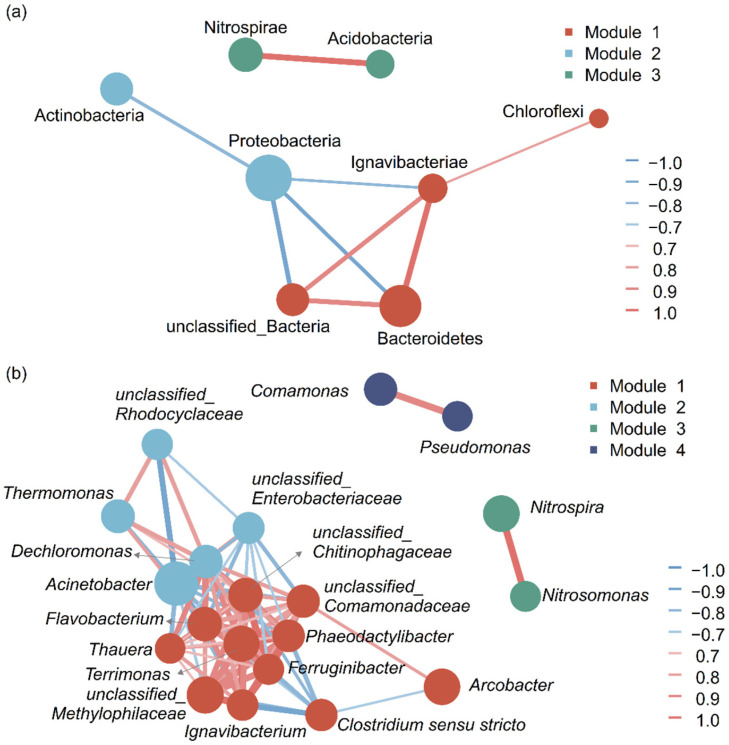
Co-occurrence network analysis for the top 10 phyla (**a**) and 20 genera (**b**) in MBBR-IFAS systems.

By constructing a combined co-occurrence network, the relationships among the top ten phyla and among the top twenty abundant genera in biofilm and activated sludge samples of MBBR-IFAS systems were further examined. At the phylum level, three modules emerged in the constructed network (Figure 5a). Module 1 included Bacteroidetes, Ignavibacteriae, and Chloroflexi; Module 2 included Proteobacteria and Actinobacteria; Module 3 included Nitrospirae and Acidobacteria. There were negative correlations between Module 1 and Module 2, where most bacteria are heterotrophic. Module 3 had no correlations with the other two modules. Acidobacteria is a phylum of acidophilic bacteria, and their co-occurrence relationship with Nitrospirae may be due to the acid-production ability of nitrifying bacteria.

At the genus level, four main modules emerged in the constructed network (Figure 5b). Module 1 included *Arcobacter*, *Terrimonas*, *Flavobacterium*, *Phaeodactylibacter*, *Clostridium sensu stricto*, *Ignavibacterium*, and *Thauera*; Module 2 included *Acinetobacter*, *Thermomonas*, *Dechloromonas*, and *Ferruginibacter*; Module 3 included *Comamonas* and *Pseudomonas*; and Module 4 included *Nitrospira* and *Nitrosomonas*. Each module appeared to be usually related to a specific function [50]. Modules 1, 2, and 3 were all dominated by heterotrophic denitrifying genera. Among these three modules, there were negative correlations between all the genera in Modules 1 and 2, and there were no correlations between Module 3 and other modules. Module 4 was dominated by nitrifying bacteria poorly connecting to other genera in the network. This phenomenon is likely due to the dependence of these genera on aerobic conditions rather than other bacteria [50], and the suspended carriers provided them with the conditions to always be in a suitable environment. In conclusion, the nitrifying bacteria seem to have a weak co-occurrence relationship with other bacteria in MBBR-IFAS systems.

This research revealed the co-occurrence relationships among the dominant bacteria in MBBR-IFAS systems.

### 3.4. Potential Functions

#### 3.4.1. Potential Functions of Pathways Based on KEGG

**Figure 6:**
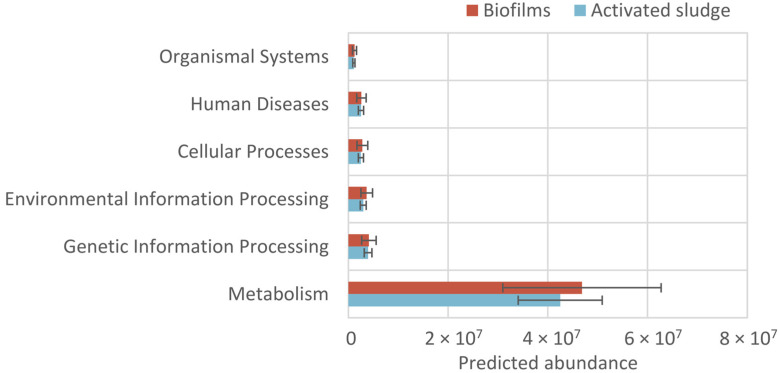
Predicted abundances for pathways-level 1 of biofilms and activated sludge in MBBR-IFAS systems based on KEGG.

There are seven pathways at level 1 (pathways-level1) based on KEGG, including metabolism, genetic information processing, environmental information processing, cellular processes, organismal systems, human diseases, and drug development. The predicted abundances of these pathways-level1 for biofilms and activated sludge in MBBR-IFAS systems are shown in Figure 6. The most abundant function was metabolism in MBBR-IFAS systems. The predicted abundance of this pathway was one order of magnitude larger than that of the rest in biofilms and activated sludge. Correspondingly, pollutant removal in wastewater treatment is mainly dependent on microbial metabolism. There were no significant differences in these seven pathways-level1 between biofilms and activated sludge.

**Figure 7:**
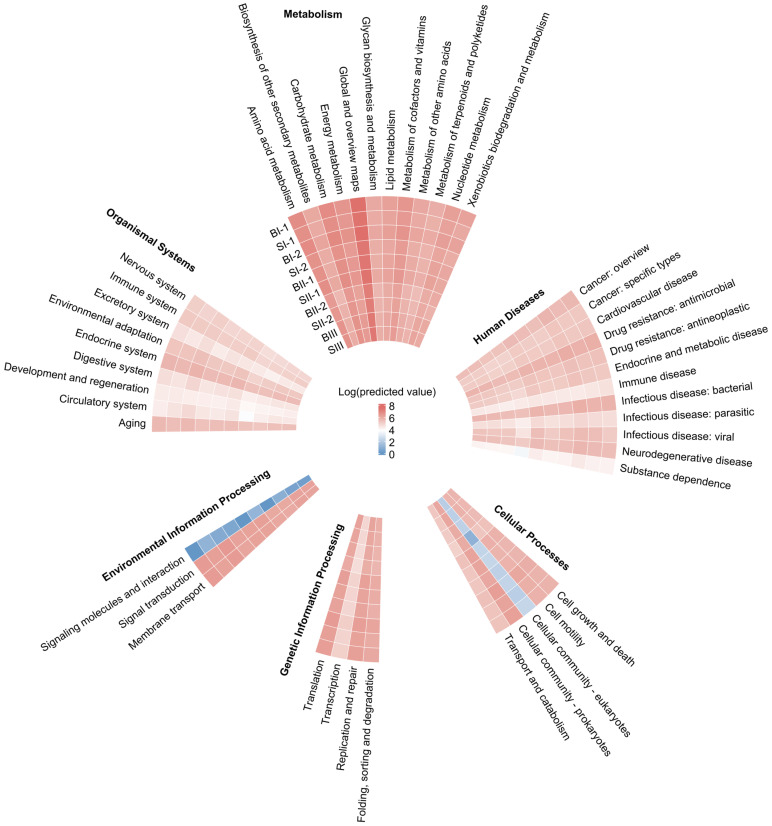
Predicted abundances for pathways-level 2 of biofilms and activated sludge in MBBR-IFAS systems based on KEGG.

The potential functions of pathways at level 2 (pathways-level2), which were members of the seven pathways-level1, are illustrated in Figure 7. The top five abundant pathways-level2 were all from metabolism: global and overview maps, carbohydrate metabolism, amino acid metabolism, energy metabolism, and metabolism of cofactors and vitamins. These functions were all associated with pollutant removals in wastewater treatment. Like pathways-level1, there were no differences in pathways-level2 between biofilms and activated sludge.

**Figure 8:**
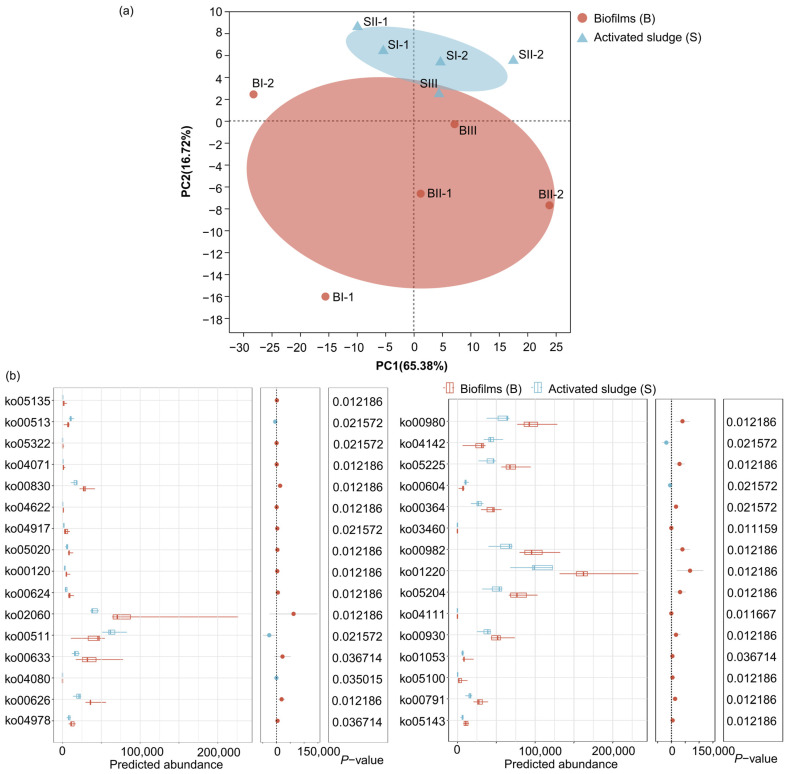
PCA (**a**) for pathways-level3 of biofilms and activated sludge and difference analysis (**b**) for pathways-level3 between biofilms and activated sludge in MBBR-IFAS systems based on KEGG.

Unlike pathway-level1 and pathway-level2, PCA for the pathways at level 3 (pathways-level3) revealed the difference between biofilms and activated sludge (Figure 8a). P1 and P2 explained 65.38% and 16.72% of variances, respectively. As a result, the samples are clustered into two groups corresponding to biofilm samples and activated sludge samples. Furthermore, the differences for pathways-level3 between biofilms and activated sludge in MBBR-IFAS systems are shown in Figure 8b. There were five pathways-level3 more significantly abundant in activated sludge than biofilms (*p* < 0.05), including various types of N-glycan biosynthesis (ko00513), other glycan degradation (ko00511), neuroactive ligand–receptor interaction (ko04080), lysosome (ko04142), glycosphingolipid biosynthesis ganglio series (ko00604). Among them, various types of N-glycan biosynthesis (ko00513), other glycan degradation (ko00511), and glycosphingolipid biosynthesis ganglio series (ko00604) belong to glycan biosynthesis and metabolism, which help stick microbial cells together and further form activated sludge. On the other hand, there were twenty-six pathways-level3 more significantly abundant in biofilms than activated sludge (*p* < 0.05). Among these pathways-level3, atrazine degradation (ko00791), biosynthesis of siderophore group nonribosomal peptides (ko01053), caprolactam degradation (ko00930), degradation of aromatic compounds (ko01220), drug metabolism-cytochrome P450 (ko00982), fluorobenzoate degradation (ko00364), metabolism of xenobiotics by cytochrome P450 (ko00980), naphthalene degradation (ko00626), nitrotoluene degradation (ko00633), polycyclic aromatic hydrocarbon degradation (ko00624), primary bile acid biosynthesis (ko00120), and retinol metabolism (ko00830) are associated with various organic biodegradation, indicating that biofilms had more potential in organic biodegradation, including removal of some micropollutants [51]. Among these pathways-level3, cell cycle–yeast (ko04111) and Fanconi anemia pathways (ko03460) are associated with cell renewal, and phosphotransferase system (PTS) (ko02060) and sphingolipid signaling pathways (ko04071) are associated with membrane and signal transportation. Higher predicted abundances of these four pathways-level3 in biofilms than in activated sludge indicated higher biomass activity [19]. In addition, the potential functions of mineral absorption (ko04978), prolactin signaling pathway (ko04917), and RIG-I-like receptor signaling pathway (ko04622) were all more abundant in biofilms than activated sludge, as well as seven pathways-level3 belonging to human diseases.

In summary, there were no significant differences between the biofilms and activated sludge of MBBR-IFAS systems on pathways-level1 and pathways-level2, but there were some differences in pathways-level3.

#### 3.4.2. Potential Functions of Modules Involved in Nitrogen Metabolism

**Figure 9:**
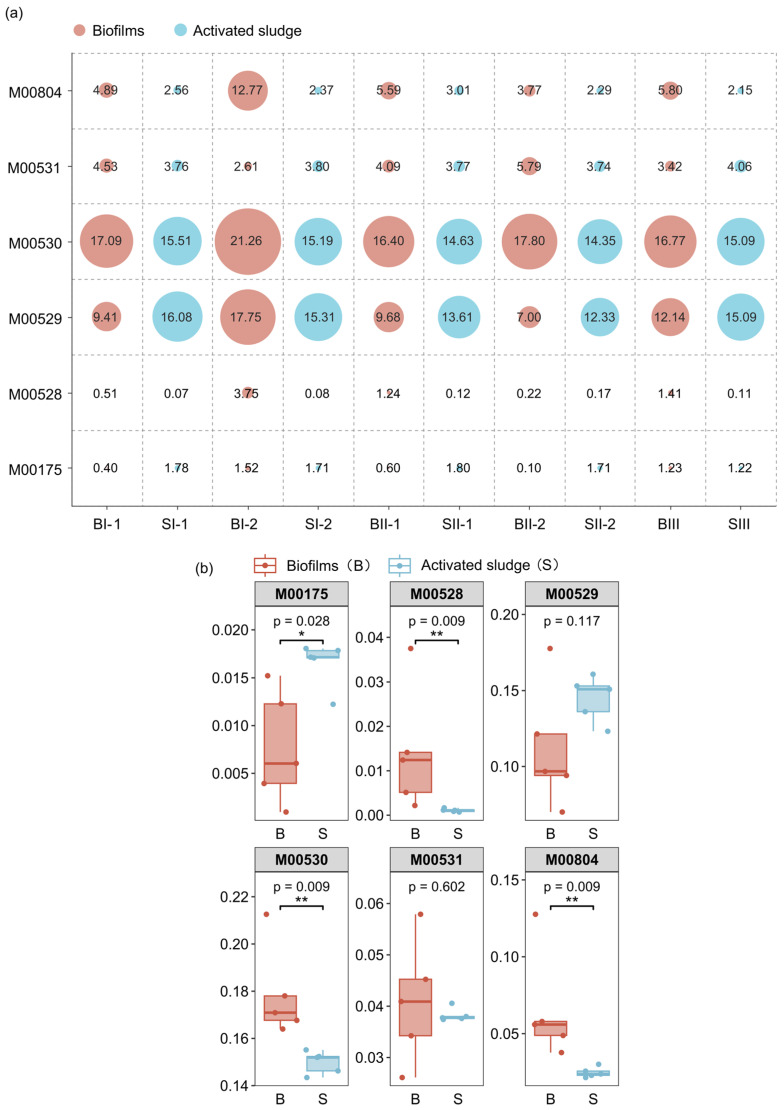
Predicted relative abundances for the core nitrogen metabolism modules of biofilms and activated sludge (**a**) and difference analysis (**b**) for the core nitrogen metabolism modules between biofilms and activated sludge in MBBR-IFAS systems. “*” presents “*p* < 0.05”, and “**” presents “*p* < 0.01”.

The potential functions of modules involved in nitrogen metabolism in MBBR-IFAS systems were explored based on KEGG (Figure 9). As shown in Figure 9a, six modules involved in nitrogen metabolism (ko00910) were predicted in oxic reactors of MBBR-IFAS systems, including nitrogen fixation (M00175), assimilatory nitrate reduction (M00531), dissimilatory nitrate reduction (M00530), denitrification (M00529), nitrification (ammonia oxidation) (M00528), and complete nitrification (comammox) (M00804). The most abundant modules of nitrogen metabolism in MBBR-IFAS systems were dissimilatory nitrate reduction (M00530) and denitrification (M00529) with average predicted relative abundances of 16.42% and 12.84%. This corresponded to the bacterial populations in the previous study. Complete nitrification and comammox (M00804) were followed by an average predicted relative abundance of 4.52%. This indicated that ammonia nitrogen removal might mainly depend on comammox in the MBBR-IFAS systems. The differences between biofilms and activated sludge in these modules were further analyzed in Figure 9b. The two modules involved in nitrification, complete nitrification (comammox) (M00804), and nitrification (ammonia oxidation) (M00528) were predicted to be more significantly abundant in biofilms than activated sludge (*p* < 0.01). This indicated that ammonia nitrogen removal was mainly performed more in biofilms than in activated sludge. This corresponded to the fact that comammox bacteria *Nitrospira* were more abundant in biofilms. In addition, dissimilatory nitrate reduction (M00530) was also more abundant in biofilms than in activated sludge. This is possibly due to the competitive effect of denitrification, as these two modules were competitive with each other due to similar substance and environmental conditions [52,53]. On the other hand, nitrogen fixation (M00175) was predicted to be more significantly abundant in activated sludge than in biofilms (*p* < 0.05).

In terms of nitrogen metabolism, biofilms had more potential functions in complete nitrification (comammox) (M00804), nitrification (ammonia oxidation) (M00528), and dissimilatory nitrate reduction (M00530) than activated sludge in the MBBR-IFAS systems.

#### 3.4.3. Potential Functions of KOs Involved in Nitrification

**Figure 10:**
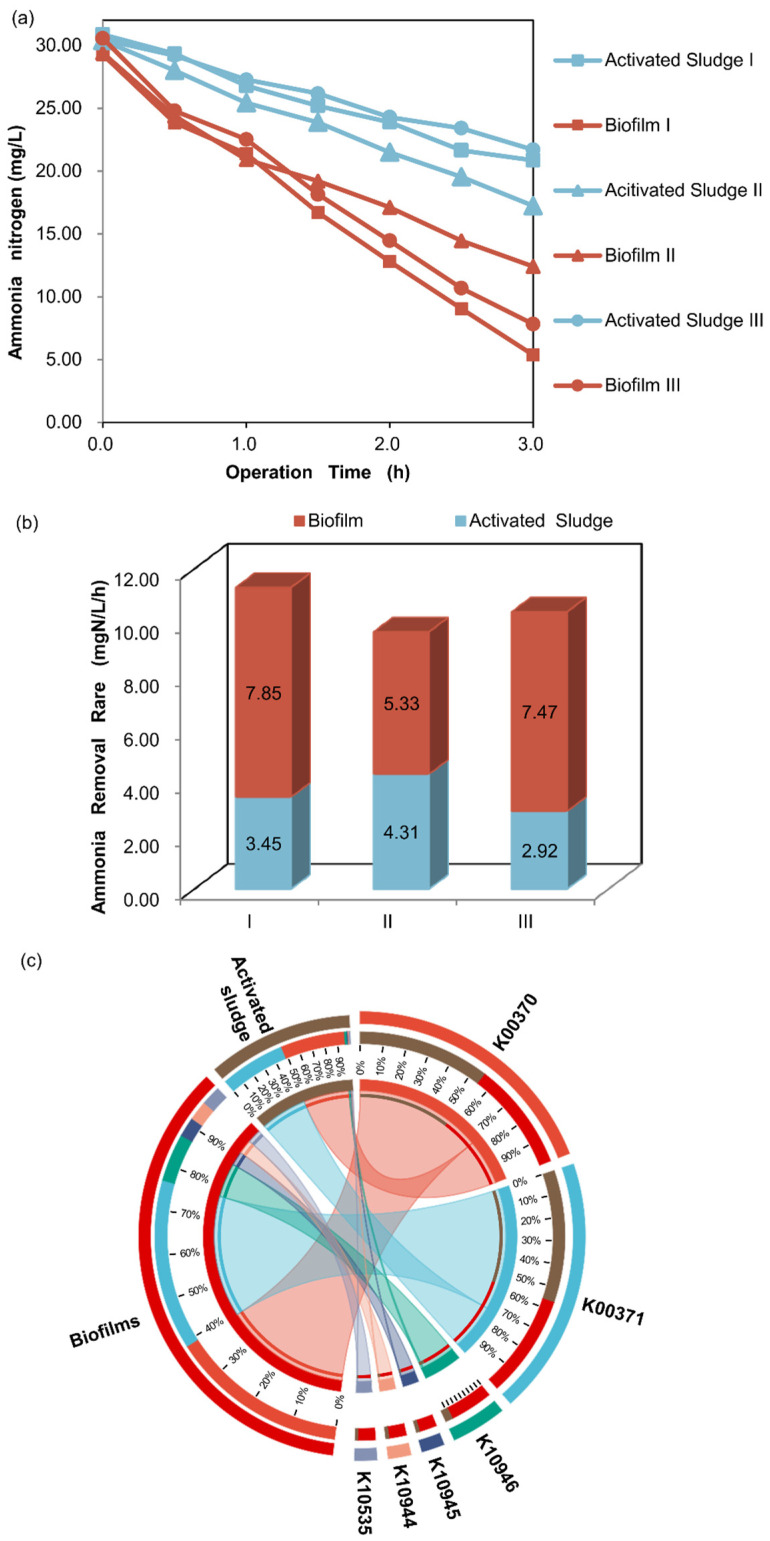
Removal effect of ammonia nitrogen (**a**), ammonia nitrogen removal rates (**b**), and predicted relative abundances of KOs involved in nitrification (**c**) of biofilms and activated sludge in MBBR-IFAS systems.

Since the main reason for adding the suspended carriers was to enhance the ammonia nitrogen removal abilities of the systems, the ammonia nitrogen removal abilities of biofilms and activated sludge in the MBBR-IFAS systems were detected (Figure 10a). The results showed that the decreasing rates of ammonia nitrogen concentration in these three MBBR systems were faster than those in the three activated sludge systems. In addition, their downward trends were consistent with the first-order reaction kinetics relationship (R^2^ > 0.97). It was calculated after linear fitting that the ammonia removal rates of biofilms were 7.85, 5.33, and 7.47 mg N/L/h, while those of activated sludge from the same MBBR-IFAS systems were only 3.45, 4.31, and 2.92 mg N/L/h, respectively (Figure 10b). Therefore, biofilms’ ammonia nitrogen removal abilities were much more potent than that of activated sludge in MBBR-IFAS systems. This is also the recognized advantage of the MBBR-IFAS systems due to the high retention time [16].

Furthermore, the potential functions of nitrification in MBBR-IFAS systems were analyzed (Figure 10c). The KOs involved in nitrification include methane/ammonia monooxygenase K10944 (pmoA-amoA), K10945 (pmoB-amoB), and K10946 (pmoC-amoC); hydroxylamine dehydrogenase K10535 (hao); and nitrate reductase/nitrite oxidoreductase K00370 (narG/narZ/nxrA) and K00371 (narH/narY/nxrB). As a result, 84%, 84%, 85%, and 86% of K10944 (pmoA-amoA), K10945 (pmoB-amoB), K10946 (pmoC-amoC), and K10535 (hao) were derived from biofilms, respectively, indicating that most of the ammonia-oxidizing potentials of the MBBR-IFAS systems came from biofilms. The relative abundances of K00370 (narG/narZ/nxrA) and K00371 (narH/narY/nxrB) in activated sludge were 47% and 48%, respectively, while they were 39% and 38% in biofilms, respectively. These two KOs symbolize nitrate reductase and nitrite oxidoreductase, which are involved in nitrification and denitrification, respectively, but not just nitrification. They were found to be abundant in both oxic and anoxic reactors [52]. So, the greater abundance of the two KOs in activated sludge than in biofilms may be due to their alternate retention in anoxic and oxic reactors.

In conclusion, biofilms have more potential functions for the KOs of methane/ammonia monooxygenase (K10944, K10945, and K10946) and hydroxylamine dehydrogenase (K10535) than activated sludge in MBBR-IFAS systems, corresponding to a higher ammonia nitrogen removal rate at the macro level.

## 4. Conclusions

As far as we know, this study is the most comprehensive exploration to date analyzing the comparisons in microbial information between biofilms and activated sludge in large, running, full-scale MBBR-IFAS municipal WWTPs. The different environments of biofilms and activated sludge in MBBR-IFAS systems inevitably form their different bacterial communities. They complement each other to achieve the goal of pollutant removal. The difference analysis results showed that some denitrifying bacteria were enriched in activated sludge, while the comammox bacteria *Nitrospira* was enriched in biofilms. Nitrifying bacteria had a weak co-occurrence relationship with other bacteria in the MBBR-IFAS systems, possibly due to the biofilm providing them with suitable habitats. PICRUSt2 analysis specific functions (pathways-level3) based on KEGG revealed that biofilms had more potential in various organic biodegradation and membrane and signal transportation. In comparison, activated sludge had more potential in glycan biosynthesis and metabolism. In terms of nitrogen metabolism, biofilms had more potential in methane/ammonia monooxygenase and hydroxylamine dehydrogenase for nitrification. This exploration of the bacterial information of the biofilms and activated sludge in MBBR-IFAS systems in this study will be a theoretical base for a practical application of the operation and design strategy of these systems.

## Figures and Tables

**Table 1:** Description of sample numbers of biofilms and activated sludge.

Sample Number	Sample Type	System Source	Reactor Source
BI-1	Biofilms	System I	The first MBBR-IFAS of System I
SI-1	Activated sludge	System I	The first MBBR-IFAS of System I
BI-2	Biofilms	System I	The second MBBR-IFAS of System I
SI-2	Activated sludge	System I	The second MBBR-IFAS of System I
BII-1	Biofilms	System II	The first MBBR-IFAS of System II
SII-1	Activated sludge	System II	The first MBBR-IFAS of System II
BII-2	Biofilms	System II	The second MBBR-IFAS of System II
SII-2	Activated sludge	System II	The second MBBR-IFAS of System II
BIII	Biofilms	System III	The MBBR-IFAS of System III

## Data Availability

The data presented in this study are available upon request to the corresponding author.

## References

[1] Almomani F. (2020). Prediction the performance of multistage moving bed biological process using artificial neural network (ANN). Sci. Total Environ..

[2] Mannina G., Capodici M., Cosenza A., Cinà P., Di Trapani D., Puglia A.M., Ekama G.A. (2017). Bacterial community structure and removal performances in IFAS-MBRs: A pilot plant case study. J. Environ. Manag..

[3] Bai Y., Zhang Y., Xie Q., Shuo C. (2016). Enhancing nitrogen removal efficiency and reducing nitrate liquor recirculation ratio by improving simultaneous nitrification and denitrification in integrated fixed-film activated sludge (IFAS) process. Water Sci. Technol..

[4] Jin Y., Ding J., Zhan W., Du J., Wang G., Pang J., Ren N., Yang S. (2023). Effect of dissolved oxygen concentration on performance and mechanism of simultaneous nitrification and denitrification in integrated fixed-film activated sludge sequencing batch reactors. Bioresour. Technol..

[5] Sander S., Behnisch J., Wagner M. (2017). Energy, cost and design aspects of coarse- and fine-bubble aeration systems in the MBBR IFAS process. Water Sci. Technol..

[6] Khudhair D.N., Hosseinzadeh M., Zwain H.M., Siadatmousavi S.M., Majdi A., Mojiri A. (2023). Upgrading the MBBR Process to Reduce Excess Sludge Production in Activated Sludge System Treating Sewage. Water.

[7] Waqas S., Bilad M.R., Man Z., Wibisono Y., Jaafar J., Indra Mahlia T.M., Khan A.L., Aslam M. (2020). Recent progress in integrated fixed-film activated sludge process for wastewater treatment: A review. J. Environ. Manag..

[8] Kwon S., Kim T.-S., Yu G.H., Jung J.-H., Park H.-D. (2010). Bacterial community composition and diversity of a full-scale integrated fixed-film activated sludge system as investigated by pyrosequencing. J. Microbiol. Biotechnol..

[9] Meng F., Guo S., Zhang L., Lu Y., Li M., Tan Y., Zha K., Yuan S. (2023). Ecological mechanisms of biofilm development in the hybrid sludge-biofilm process: Implications for process start-up and optimization. Water Res..

[10] Zhang K., Li J., Zheng Z., Zhang J., Sun M., Huang S. (2022). Analyzing the sludge characteristics and microbial communities of biofilm and activated sludge in the partial nitrification/anammox process. J. Water Process Eng..

[11] Li J., Li A., Li Y., Cai M., Luo G., Wu Y., Tian Y., Xing L., Zhang Q. (2022). PICRUSt2 functionally predicts organic compounds degradation and sulfate reduction pathways in an acidogenic bioreactor. Front. Environ. Sci. Eng..

[12] Zhao J., Li Y., Euverink G.J.W. (2022). Effect of bioaugmentation combined with activated charcoal on the mitigation of volatile fatty acids inhibition during anaerobic digestion. Chem. Eng. J..

[13] Douglas G.M., Maffei V.J., Zaneveld J.R., Yurgel S.N., Brown J.R., Taylor C.M., Huttenhower C., Langille M.G. (2020). PICRUSt2 for prediction of metagenome functions. Nat. Biotechnol..

[14] Cayetano R.D.A., Park J., Kim G.-B., Jung J.-H., Kim S.-H. (2021). Enhanced anaerobic digestion of waste-activated sludge via bioaugmentation strategy—Phylogenetic investigation of communities by reconstruction of unobserved states (PICRUSt2) analysis through hydrolytic enzymes and possible linkage to system performance. Bioresour. Technol..

[15] Yang C., Mai J., Cao X., Burberry A., Cominelli F., Zhang L. (2023). ggpicrust2: An R package for PICRUSt2 predicted functional profile analysis and visualization. Bioinformatics.

[16] Giorgio M., Marco C., Alida C., Daniele D.T., Zhengyu Z., Yongmei L. (2020). Integrated Fixed Film Activated Sludge (IFAS) membrane BioReactor: The influence of the operational parameters. Bioresour. Technol..

[17] Huang M., Zhang W., Liu C., Hu H. (2014). Fate of trace tetracycline with resistant bacteria and resistance genes in an improved AAO wastewater treatment plant. Process Saf. Environ. Prot..

[18] Ashrafi E., Mehrabani Zeinabad A., Borghei S.M., Torresi E., Muñoz Sierra J. (2019). Optimising nutrient removal of a hybrid five-stage Bardenpho and moving bed biofilm reactor process using response surface methodology. J. Environ. Chem. Eng..

[19] Water Environment Federation (2010). Biofilm Reactors.

[20] Ping L., Bingyu D., Chunyan F., Shuwei Y., Ting Z., Xin Q., Huiying L., Xiaokui G., Ke D., Zhu Y. (2017). Prevotella and Klebsiella proportions in fecal microbial communities are potential characteristic parameters for patients with major depressive disorder. J. Affect. Disord..

[21] Yan K., Jian Z., Huijun X., Zizhang G., Ngo H.H., Wenshan G., Shuang L. (2017). Enhanced nutrient removal and mechanisms study in benthic fauna added surface-flow constructed wetlands: The role of Tubifex tubifex. Bioresour. Technol..

[22] Ren Y., Yu G., Shi C., Liu L., Guo Q., Han C., Zhang D., Zhang L., Liu B., Gao H. (2022). Majorbio Cloud: A one-stop, comprehensive bioinformatic platform for multiomics analyses. iMeta.

[23] Pang H.L., Xu Y.M., Ren R.Y., He J.G., Pan X.L., Wang L. (2023). Enhanced anaerobic digestion of waste activated sludge by alkaline protease-catalyzing hydrolysis: Role and significance of initial pH adjustment. Chem. Eng. J..

[24] Walter W.G. (1961). Standard Methods for the Examination of Water and Wastewater.

[25] Xu N., Tan G.C., Wang H.Y., Gai X.P. (2016). Effect of biochar additions to soil on nitrogen leaching, microbial biomass and bacterial community structure. Eur. J. Soil Biol..

[26] Wang Y., Sheng H.F., He Y., Wu J.Y., Jiang Y.X., Tam N.F.Y., Zhou H.W. (2012). Comparison of the Levels of Bacterial Diversity in Freshwater, Intertidal Wetland, and Marine Sediments by Using Millions of Illumina Tags. Appl. Environ. Microbiol..

[27] Simpson E.H. (1949). Measurement of diversity. Nature.

[28] Herrera A.M., Riera R., Rodríguez R.A. (2023). Alpha species diversity measured by Shannon’s H-index: Some misunderstandings and underexplored traits, and its key role in exploring the trophodynamic stability of dynamic multiscapes. Ecol. Indic..

[29] Liu H., Zhu H., Xia H., Yang X., Yang L., Wang S., Wen J., Sun G. (2021). Different effects of high-fat diets rich in different oils on lipids metabolism, oxidative stress and gut microbiota. Food Res. Int..

[30] Shi Y., Li W., Boyao Z., Yao G., Shi X. (2022). Characteristics of Bacterial Community Structure in Wuliangsu Lake During an Irrigation Interval in Hetao Plain. Environ. Sci..

[31] Zhao W., Peng Y., Wang M., Huang Y., Li X. (2019). Nutrient removal and microbial community structure variation in the two-sludge system treating low carbon/nitrogen domestic wastewater. Bioresour. Technol..

[32] Wang X., Bi X., Hem L.J., Ratnaweera H. (2018). Microbial community composition of a multi-stage moving bed biofilm reactor and its interaction with kinetic model parameters estimation. J. Environ. Manag..

[33] Fan X., Gao J., Pan K., Li D., Dai H., Li X. (2018). Functional genera, potential pathogens and predicted antibiotic resistance genes in 16 full-scale wastewater treatment plants treating different types of wastewater. Bioresour. Technol..

[34] Lee I.S., Parameswaran P., Rittmann B.E. (2011). Effects of solids retention time on methanogenesis in anaerobic digestion of thickened mixed sludge. Bioresour. Technol..

[35] Podosokorskaya O.A., Kadnikov V.V., Gavrilov S.N., Mardanov A.V., Merkel A.Y., Karnachuk O.V., Ravin N.V., Bonch-Osmolovskaya E.A., Kublanov I.V. (2013). Characterization of Melioribacter roseus gen. nov., sp nov., a novel facultatively anaerobic thermophilic cellulolytic bacterium from the class Ignavibacteria, and a proposal of a novel bacterial phylum Ignavibacteriae. Environ. Microbiol..

[36] Li W.C., Niu Q.G., Zhang H., Tian Z., Zhang Y., Gao Y.X., Li Y.Y., Nishimura O., Yang M. (2015). UASB treatment of chemical synthesis-based pharmaceutical wastewater containing rich organic sulfur compounds and sulfate and associated microbial characteristics. Chem. Eng. J..

[37] Song J.Y., Wu H.Y., Yuan H.M., Wu J., Qi W.K., Lu J.B., Li W.C., Li Y.Y. (2022). Reclamation of pharmaceutical wastewater by UAF, contact oxidation, and auto brush-cleaning aiding nano ZrO_2_ coated ceramic membrane MBR—an industrial-scale practice and functional microbial diversity. J. Water Process Eng..

[38] Wang K.M., Zhou L.X., Ji K.F., Xu S.N., Wang J.D. (2022). Evaluation of a modified internal circulation (MIC) anaerobic reactor for real antibiotic pharmaceutical wastewater treatment: Process performance, microbial community and antibiotic resistance genes evolutions. J. Water Process Eng..

[39] Guo X., Li B., Zhao R., Zhang J., Lin L., Zhang G., Li R.-H., Liu J., Li P., Li Y. (2019). Performance and bacterial community of moving bed biofilm reactors with various biocarriers treating primary wastewater effluent with a low organic strength and low C/N ratio. Bioresour. Technol..

[40] Han F., Ye W., Wei D., Xu W., Du B., Wei Q. (2018). Simultaneous nitrification-denitrification and membrane fouling alleviation in a submerged biofilm membrane bioreactor with coupling of sponge and biodegradable PBS carrier. Bioresour. Technol..

[41] Zhao B., He Y.L., Hughes J., Zhang X.F. (2010). Heterotrophic nitrogen removal by a newly isolated Acinetobacter calcoaceticus HNR. Bioresour. Technol..

[42] Cao L., Ni L., Qi L., Wen H.T., Wang Z., Meng J.Q., Zhang X.B., Zhang Y.F. (2023). The application of post-denitrification fixed biofilm reactor for polishing secondary effluent: Nitrate removal, soluble microbial products and micropollutants biotransformation. Bioresour. Technol..

[43] Chen H., Zhao X., Cheng Y., Jiang M., Li X., Xue G. (2018). Iron robustly stimulates simultaneous nitrification and denitrification under aerobic conditions. Environ. Sci. Technol..

[44] Huang C., Yuan L., Niu W., Meng Y. (2021). Effect of dosing suspended fillers on microbial community structure and investigation on variation in water quality. China Environ. Sci..

[45] Pishgar R., Dominic J.A., Sheng Z.Y., Tay J.H. (2019). Denitrification performance and microbial versatility in response to different selection pressures. Bioresour. Technol..

[46] Zhang X., Chi Y., Wang Q., Jin X., Shi X., Jin P. (2020). Effects of aeration strategy on denitrifying performance of activated sludge processes in treating low-carbon-source municipal wastewater. Environ. Sci..

[47] Wang D.P., Meng Y.B., Meng F.G. (2022). Genome-centric metagenomics insights into functional divergence and horizontal gene transfer of denitrifying bacteria in anammox consortia. Water Res..

[48] Wang Z.C., Yuan S.Y., Deng Z.W., Wang Y.J., Deng S.L., Song Y.T., Sun C.T., Bu N.S., Wang X.R. (2020). Evaluating responses of nitrification and denitrification to the co-selective pressure of divalent zinc and tetracycline based on resistance genes changes. Bioresour. Technol..

[49] Roots P., Wang Y., Rosenthal A.F., Griffin J.S., Sabba F., Petrovich M., Yang F., Zhang H., Wells G.F. (2019). Comammox Nitrospira are the dominant ammonia oxidizers in a mainstream low dissolved oxygen nitrification reactor. Water Res..

[50] Xiong W., Jousset A., Guo S., Karlsson I., Zhao Q.Y., Wu H.S., Kowalchuk G.A., Shen Q.R., Li R., Geisen S. (2018). Soil protist communities form a dynamic hub in the soil microbiome. Isme J..

[51] Luo Y.L., Jiang Q., Ngo H.H., Nghiem L.D., Hai F.I., Price W.E., Wang J., Guo W.S. (2015). Evaluation of micropollutant removal and fouling reduction in a hybrid moving bed biofilm reactor-membrane bioreactor system. Bioresour. Technol..

[52] Zhou X., Bi X., Yang T., Fan X., Shi X., Wang L., Zhang Y., Cheng L., Zhao F., Maletskyi Z. (2022). Metagenomic insights into microbial nitrogen metabolism in two-stage anoxic/oxic-moving bed biofilm reactor system with multiple chambers for municipal wastewater treatment. Bioresour. Technol..

[53] Preena P.G., Kumar V.J.R., Singh I.S.B. (2021). Nitrification and denitrification in recirculating aquaculture systems: The processes and players. Rev. Aquac..

